# Evaluation of the Cadmium Accumulation in Tamarillo Cells (*Solanum betaceum*) by Indirect Electrochemical Detection of Cysteine-Rich Peptides

**DOI:** 10.3390/molecules24122196

**Published:** 2019-06-12

**Authors:** Marjorie Montero-Jiménez, Lenys Fernández, José Alvarado, Mauricio Criollo, Mónica Jadán, David Chuquer, Patricio Espinoza-Montero

**Affiliations:** 1 Escuela de Ciencias Químicas, Pontificia Universidad Católica del Ecuador, Avenida 12 de Octubre y Roca, 17-01-2184 Apartado, Quito, Ecuador; marjorie_cpp@hotmail.com (M.M.-J.); dchuquer295@puce.edu.ec (D.C.); 2 Departamento de Química, Universidad Simón Bolívar, 89000 Apartado, Caracas, Venezuela; jalvar@usb.ve; 3 Centro de Investigación y Control Ambiental “CICAM”, Departamento de Ingeniería Civil y Ambiental, Facultad de Ingeniería Civil y Ambiental, Escuela Politécnica Nacional, Ladrón de Guevara E11-253, 17-01-2759 Quito, Ecuador; mauricio.criollo@epn.edu.ec; 4 Grupo BIOCEMP, Laboratorio de Cultivo de Tejidos Vegetales, Departamento de Ciencias de la Vida y de la Agricultura, Universidad de las Fuerzas Armadas ESPE, Av. General Rumiñshui s/n, 171-5-231B Sangolqui, Ecuador; mbjadan@espe.edu.ec

**Keywords:** cysteine-rich peptides, electrochemical determination, cadmium contamination, *Solanum betaceum* as an indicator

## Abstract

Long-term cadmium intake can be very dangerous to human health due to its toxic effects. Although people can be contaminated with this element from different sources, contaminated food is probably the most important one. Foods such as vegetables and fruits can become contaminated with cadmium existing in soils, irrigation water, or chemical fertilizers. Some plants produce an excess of cysteine-rich peptides (CRp) when affected by high concentrations of heavy metals such as cadmium, thus indicating the presence of this type of contamination. Among these plants is tamarillo (*Solanum betaceum*), which is locally known as “tree tomato”. This is a native plant widely consumed in the Ecuadorian Andes because of its abundance, low cost, and high content of vitamin C and fiber. The fact that *Solanum betaceum* produces CRp upon contamination with heavy metals means that this plant may be able to accumulate heavy metals. If this is the case, the plant can possibly be used as an indicator of metal pollution. The main goals of the present work were to evaluate the possibility of using *Solanum betaceum* as an indicator of metal contamination in plants and to examine its capability to accumulate metals. Both goals were met by determination of the amounts of CRp produced by *Solanum betaceum* cells cultivated in vitro in the laboratory under controlled conditions in the presence of different concentrations of cadmium. The CRp determination was carried out by means of electrogeneration of iodine in an iodide solution containing reduced glutathione as a biological thiol model. *Solanum betaceum* cells were grown in a Murashige and Skoog solution enriched with a 30 g L^−1^ sugar aqueous solution and 1 mg L^−1^ 2,4-dichlorophenoxyacetic acid. The results of these experiments confirmed the following: (1) CRp production is a function of the amount of cadmium present as a contaminant up to a limiting value after which cell apoptosis occurs; (2) *Solanum betaceum* accumulates cadmium; (3) the analytical method used is appropriate for CRp determination; and (4) CRp determination is a valid alternative to detect contamination by heavy metals in plants.

## 1. Introduction

Long-term intake of foods contaminated with Cd, Pb, Hg, and some other heavy metals can result in serious health problems due to their accumulative character and toxicity [1,2]. Cadmium is an element that can be present in the water, soil, pesticides, and chemical fertilizers. It can be considered as one of the most ubiquitous toxic heavy metals in plants because this element can be easily absorbed through the roots, accumulated, and stored in the whole plant. In the case of edible plants, this metal can be transferred to people through the trophic chain, constituting a health risk. The presence of this contaminant in crops used as sources of food for human beings should therefore be closely monitored [3,4]. The presence of excess heavy metals in a given plant can be detected by measuring the amount of cysteine-rich peptides (CRp), such as phytochelatins and glutathione (GSH), produced by some plants when they are under stress caused by the presence of heavy metals. It seems that CRp production is some sort of a defense mechanism of some plants in order to minimize perturbation of its intracellular equilibria due to the presence of heavy metals. These equilibria allow genetic signal transduction, plant metabolism, and gene regulation to continue functioning properly [4,5]. There are several methods of detecting CRp, including those based on fluorescence [6], chromatography [7], colorimetry [8], and mass spectroscopy [9]. However, these methods require lengthy and laborious sample treatments as well as high analysis costs [10,11]. Electrochemical methods for CRp determination have been well accepted as an alternative to the previously mentioned methods. Electrochemical methods are highly sensitive, produce results in comparatively shorter times, and require lower costs [12]. However, to the best of our knowledge, there have not been any published articles in the specialized literature dealing with CRp generation by *Solanum betaceum*, also known as “tomato tree” in Ecuador, other South American countries, and some parts of Europe. In Ecuador, the fruit of *Solanum betaceum* (tomato tree) is heavily commercialized in most cities and consumed by many people because of its great taste, nutritional qualities, and low cost [13,14,15]. The fruit looks like a standard tomato, although it is smaller in size, and this could be the reason the fruit is locally known as “tomato tree”. The fruit can be eaten on its own or as a salad ingredient. Unfortunately, the lack of control over the use of chemical pesticides, fertilizers, and, in some instances, contaminated water for soil and plant irrigation can result in contamination of fruits and edible vegetables with several toxic species, including heavy metals [16]. In the present work, we deal with the evaluation of *Solanum betaceum* as a possible indicator of cadmium contamination as assessed by the determination of CRp produced by the plant as a response to the presence of such contaminants. The work is aimed at the indirect quantification of CRp in *Solanum betaceum* cells grown in vitro in the presence of cytotoxic levels of cadmium following electrogenerated iodine reaction in an electrolytic solution of iodide ions, with reduced glutathione (RSH) used as a thiol model. Iodine is a selective reagent for oxidation of RSH to glutathione disulfide (GSSG) according to the following reactions, which can be monitored by means of electrochemical techniques [17,18,19]

(1) $$2I^{-}\left(\mathrm{aq}\right)⇄I^{-2}\left(\mathrm{aq}\right)+2e^{-}$$

(2) $$I^{-2}\left(\mathrm{aq}\right)⇄I_{2}\left(s\right)$$

(3) $$2\mathrm{RSH}+I_{2}\overset{I^{-}}{\to }\mathrm{RSSR}+2\mathrm{HI}$$

If the above reactions are followed by means of cyclic voltammetry (CV), the voltammograms would show a reversible process characterized by the presence of oxidation and reduction peak signals due to the electron transference occurring during the reaction expressed by Equation (1) above. When the RSH peptide is gradually and continuously added to the solution, Equation (3) becomes important, and the oxidation peak increases while the reduction peak decreases in accordance with what would be expected from a catalytic process [18,20]. The increase in the oxidation peak as a function of the RSH added is effectively used as an indirect way to ascertain the amount of peptide present in the system. Electrochemical determination of cysteine-rich peptides generated by plants under stress by the presence of Cd(II) using the iodine method has been previously applied to different types of plants. Cadmium determination in pea (*Pisum sativum*) leaves was carried out by Gonzalez et al. [18], while Fojta used the method to study Cd(II) contamination in tobacco leaves [21]. Quantitation of CRp in the *Solanum betaceum* plant would be an efficient way of determining if crops are contaminated with heavy metals in order to take appropriate measures to prevent consumers from their intake. Tamarillo can also be strategically planted along with some other edible species to be used as an indicator of contamination of the rest of the plants. The results of the analytical method used to determine heavy metal contamination in the tomato tree itself can, by extension, indicate possible contamination of the rest of the plants. We initiated the present research studying the production of CRp in cells of *Solanum betaceum* cultivated in the laboratory because, through this way, we were able to control conditions such as the number of cells, nutrients, humidity, temperature, and some other conditions that could favor cell growth, transplantation, and their development into fully mature plants. We could also have an insight into the metabolic behavior of the cells, the way they develop, the time needed for full maturity, and the efficiency of CRp generation as a function of the plant´s growth [22]. The information gathered from this study will be a valuable tool for seeding and cultivating plants of *Solanum betaceum* with the purpose of determining whether their fruits are contaminated with heavy metals or even to know if this plant can be used as an indicator of heavy metal contamination of a given crop used as a source of food. Despite the fact that *Solanum betaceum* grows wildly under the climatologic conditions of most South American Andean soils, the knowledge acquired from the present work will be useful in enhancing and maximizing the growth of this plant and to open up a new and important use of this plant as an indicator of metal pollution in other crops.

## 2. Results

### 2.1. Germination of Cell Extracts

**Figure 1:**
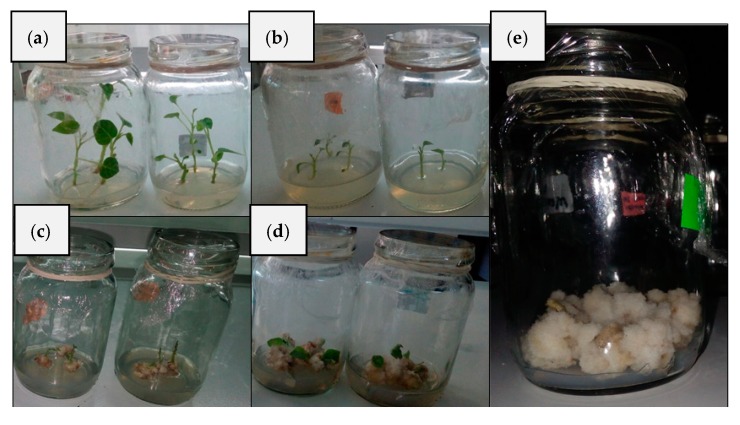
*Solanum betaceum*: (**a**) germinated plants, (**b**) callus planting, (**c**) two-week-old callus, (**d**) four-week-old callus, (**e**) four-week-old tobacco callus.

Figure 1 shows the results obtained from the in vitro experiments. Seeds were germinated six weeks after planting (Figure 1a). Replication of sprouts was performed six weeks after germination (Figure 1b), and replication assays were carried out every four weeks. The embryogenic callus and agglomerations of undifferentiated cells are shown in Figure 1c,d. Callus obtained from the leaves was smaller than those obtained from the stems but the former lasted longer than the latter, i.e., six and four weeks, respectively. Table 1 shows the percentage of explants that were able to generate callus as a function of the treatment received. Statistically, with a 95% confidence level, the best treatments applied were the ones based on the addition of 1 mg L^−1^ of 2,4-dichlorophenoxyacetic acid (2,4-D) for the stem explants and the addition of 5 mg L^−1^ 1-naphthaleneacetic acid (ANA) for the leaf explants. Therefore, we decided to continue the work with four-week-old stem explants in the Murashige and Skoog (MS) medium supplemented with 30 g L^−1^ sugar plus 1 mg L^−1^ 2,4-D. Prior to analysis, the callus suspended in the MS solution was subjected to mechanical shaking for two weeks. With the purpose of avoiding the cell death and to eliminate agglomerated cells, a representative subculture was initiated in a fresh medium [23].

**Figure 2:**
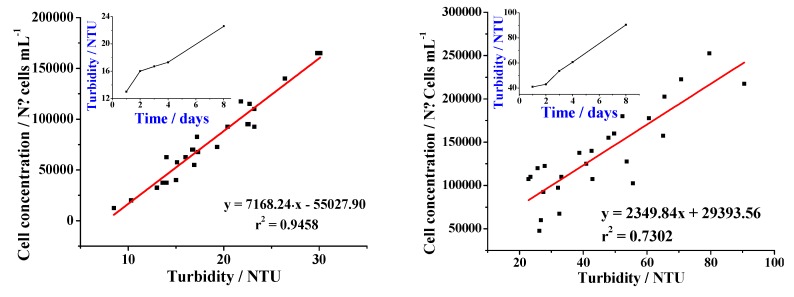
Cell concentration versus suspension turbidity.

**Figure 3:**
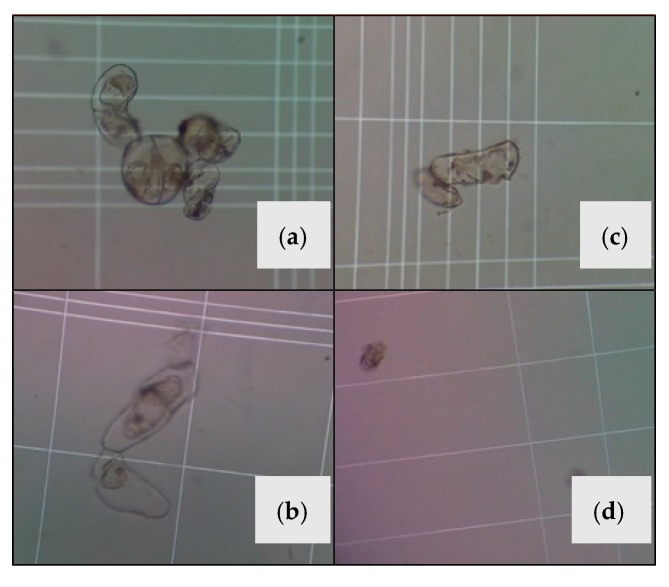
Microscopic (10X) views of cell suspensions of *Solanum betaceum*: (**a**) large groups, (**b**,**c**) smaller groups, and (**d**) completely dispersed cells.

Figure 2 shows the cell growth in a period of eight days as measured with the Neubauer camera. It is clear that cell growth and suspension turbidity were directly related. According to the inset, turbidity increased as a function of time, indicating that cell growth was still ongoing after eight days. This attested to the effectiveness of the 30 g L^−1^ sugar and 1 mg L^−1^ 2,4-D MS solution as a very appropriate medium for cell growth. According to Delgado et al. [24], when cell growth stops, cell number remains stationary for a brief period of time and then decreases due to the cell death. As indicated in Figure 2, cells in the suspension did not reach the stationary phase in the time period used in this study, i.e., there was no cell death and therefore there was no need for any additional subculture. Figure 3 shows images of cells seen through an optical microscope under a 10X enhancement. It can be easily seen that the cells are totally disaggregated, which makes these suspensions useful for monitoring cell growth.

### 2.2. Evaluation of the Analytical Method

**Figure 4:**
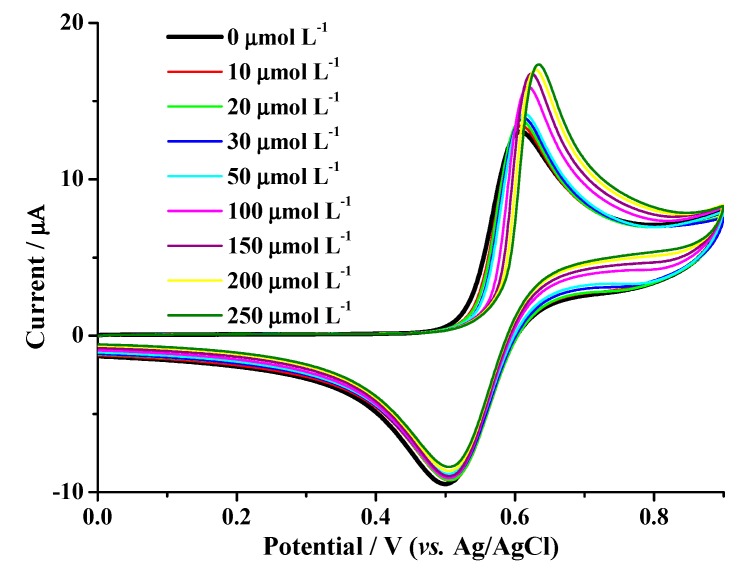
Cyclic voltammetry of a 1 mmol L^−1^ KI solution in the presence of different amounts of reduced glutathione. Scan speed, 0.05 V s^−1^; Pt used as working electrode, graphite used as counter electrode.

**Figure 5:**
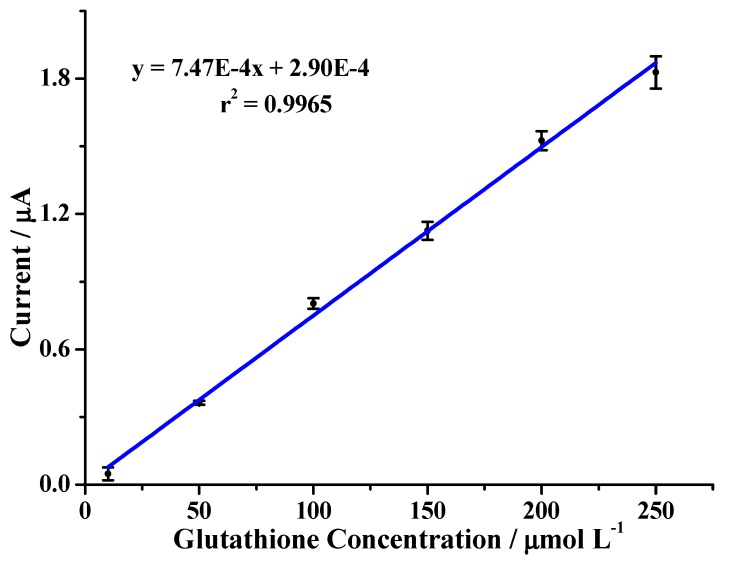
Calibration curve for reduced glutathione on Pt working electrode.

Figure 4 shows the cyclic voltammetry curves using a Pt electrode prepared in accordance with cleaning method 5, as described in the experimental section, of a 1 mmol L^−1^ KI solution in the absence (black line) and the presence (other colors) of different amounts of reduced glutathione. The oxidation peak at 0.6 V, corresponding to the I^−^ to I_2_ transformation, increased as a function of GSH addition, while the reduction peak at 0.5 V decreased due to the catalytic process of the reagent in the KI medium, which made it possible to oxidize the peptide through its reaction with the electrochemically generated I_2_ species. In aqueous solutions, the I_2_ produced reacts with the thiol groups in the peptide molecules to convert them into disulfide groups according to the following reaction 2RSH + I_2_ → RSSR + 2HI. The difference between the current values for oxidation with and without the peptide was taken to determine the concentration of the peptide using the curve depicted in Figure 5.

Figure 5 shows the calibration curve obtained at the platinum electrode using the best cleaning treatment for the electrode. The best coefficients of determination were obtained by polishing the working electrode mechanically. Afterward, electrochemical cleaning was performed, and a blank was measured before each measurement for the best treatment (method 5). Table 2 presents the variation coefficient of repeatability and reproducibility analysis and the determination coefficient. The resulting values satisfied the acceptance criteria.

Table 2 refers to the reproducibility and repeatability coefficients of variation of the values used to build the calibration curve shown in Figure 5 as well as the linearity coefficient r^2^ of the curve. All values were within the limits usually accepted for a good working curve. Under the analytical conditions observed for building the calibration curve, the method provided a detection limit of 4.82 µmol L^−1^ (3.3 σ/m), a quantitation limit of 15.41 µmol L^−1^ (10σ/m), and a sensitivity of 7.46 × 10^−4^ Aµ L µmol. Other experiments aimed at obtaining calibration curves were also performed using the same Pt electrode but after different cleaning processes. The best results, used to build the curve in Figure 5, were obtained after cleaning the electrode according to method 5. The need for cleaning the electrode after each reading is due to the fact that KI can degrade with time, causing different measurements during lengthy experiments [25]. KI degradation can result in different iodide concentrations in the electrode surface during the first measurements compared with the later ones. This would lead to a decrease in the oxidation peaks that do not correspond with different concentrations of the peptides in the solution, therefore leading to an error in the peptide determination.

### 2.3. Measurement of Peptides in Cell Suspensions

**Figure 6:**
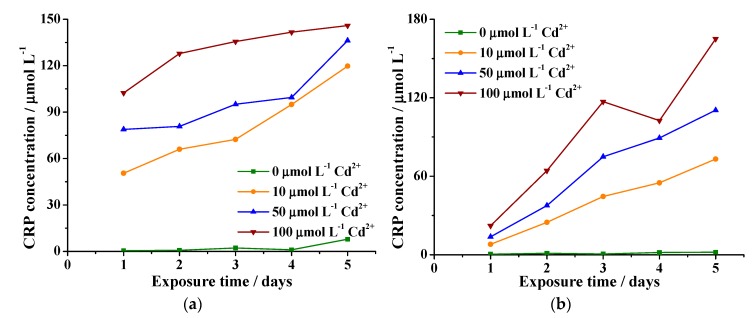
The relationship between cysteine-rich peptide (CRp) concentration and exposure time to different cadmium sulfate concentrations in cells of (**a**) tamarillo and (**b**) tobacco.

Experiments were carried out in cell suspensions with a turbidity of 87.8 NTU, which means a cell density of around 574,344 cells mL^−1^ (Figure 2). Figure 6 shows the relationship between CRp generation and exposition time of cells of (a) *Solanum betaceum* and (b) tobacco to different CdSO_4_ concentrations. The standard addition method was used to determine the CRp values in order to minimize the possible dependence of the signals on the sample´s matrix [27]. It is clear from Figure 6a,b that CRp values were practically zero in the absence of cadmium up to day 5 of the experiment, indicating that there was no contamination or any other factor that could cause stress on the cells. For the tomato tree´s cells, the small increase in the CRp concentration after day 4 could be attributed to consumption of the culture medium containing the peptide.

From Figure 6a, it is evident that, in general terms, the CRp concentration increased over time up to day 5 after exposure to cadmium. As expected, the lesser amount of cadmium added, i.e., 10 µmol·L^−1^, resulted in lesser deviations from linearity as a function of time. Increasing the amount of cadmium added to 50 µmol·L^−1^ caused a small change in slope at day 3 but with no significant reduction on the generation of CRp. Addition of the 100 µmol·L^−1^ Cd solution resulted in a continuous increase in the curve, with no drastic changes in the slope even up to day 5. This behavior discards the possibility of tamarillo cells undergoing apoptosis under these contamination conditions and exposure times [21]. Looking at Figure 6b and according to Fojta et al., tobacco cells experience apoptosis on the fifth day of exposure to 100 µmol·L^−1^ CdSO_4_. It has been reported that prior to cell death, a maximal amount of CRp is produced due to the maximal stress caused to the cells by the heavy metal contaminant [21]. When the heavy metal gets inside the cell structure of the plant, a defense mechanism is activated and CRp is generated. In these peptides, metals react with their thiol groups to form a metal−organic complex, which is transported to the vacuoles and bioaccumulated inside the cells [28,29]. Our results demonstrate that *Solanum betaceum* cells accumulate as much as or even more Cd than the tobacco cells with the added advantage of higher tolerance to contamination.

## 3. Materials and Methods 

### 3.1. Materials

Tamarillo seeds were obtained from Granja Tumbaco del INIAP (Instituto Nacional de Investigadores Agropecuarios, Quito, Ecuador). All reagents were analytical grade. Solutions were prepared using ultrapure water 18 MΩ cm^−1^, Bacto Agar (Biomark, Dhairi, India). Macronutrients: CaCl_2_ (Fisher Chemical, Waltham, MA USA, 100%), KH_2_PO_4_ (Himedia, Mumbai, India, 99%), KNO_3_ (Merck, Darmstadt, Germany, 99%) MgSO_4_·7H_2_O (Mallinckrodt Chemicals, Dublin, Ireland, 100%), and NH_4_NO_3_ (Merck, Darmstadt, Germany, 98.5%). Micronutrients: CoCl_2_·6H_2_O (Sigma, Darmstadt, Germany, 100%), CuSO_4_·5H_2_O (Himedia, Mumbai, India, 99.5%), FeNaEDTA (Sigma, Darmstadt, Germany, 99%), H_3_BO_3_ (Fisher, Waltham, MA USA, 99.8%), KI (Fisher Scientific, Waltham, MA USA, 99.5%), MnSO_4_·H_2_O (Himedia, 98%), Na_2_MoO_4_·2H_2_O (Fisher Chemicals, 99.8%), and ZnSO_4_·7H_2_O (Lobachemie, Mumbai, India, 99.5%). Vitamins: glicine (Sigma, 99%), mio-inositol (Lobachemie, 99%), nicotinic acid (Lobachemie, 99.5%), piridoxine HCl (Sigma, 99%), and tiamine HCl (Lobachemie, 99%). Fitohormons: 2,4-dichlorophenoxyacetic acid (Loba Chemie, 98%) and 1-naphthaleneacetic acid (Lobachemie, 99). Electrolytic media: KI (HVO, Crater Rim Drive, HI, USA, 100%), H_2_SO_4_ (Merck, 98%), K_2_SO_4_ (Sigma, >98%), reduced L-glutathion (Sigma, >98%), NaCl (Baker, Sanford, ME, USA, 100%), KCl (Merck, >99.5%), ethanol (Fisher, 99.8%), CdSO_4_ (Merck, 98%), and Na_2_HPO_4_ (Fisher Scientific, 100%).

The following equipment were used for culturing the *Solanum betaceum* cells: pH meter (Thermo Scientific, Waltham, MA, USA); laminar flow box (Esco, Horsham, UK); microscope (Olympus BX-41, Tokyo, Japan); autoclave (Tuttnauer, Chicago, IL, USA), and orbital shaker (WiseShake, Lutterworth, UK).

The following were used for evaluation of the analytical protocol and CRp quantitation: potentiostat (CH Instruments, Austin, TX, USA) with a platinum working electrode, an Ag/AgCl reference electrode, and a graphite counter electrode; ultrasonic bath (Branson, MI, USA); field turbidimeter (Hach, Loveland, CO, USA); and centrifuge (Hermle, Gosheim, Germany).

### 3.2. Preparation of Cell Extracts

Seeds were extracted from the fruits and allowed to dry for a period of one month inside a laminar flow clean box. Seeding of the cells, cell sprouting, preparation of cell suspensions, subculture, and contamination with CdSO_4_ were all carried out inside a laminar box previously disinfected with a 70% ethanol aqueous solution and exposed to UV radiation for 40 min [30].

The *Solanum betaceum* seeds were disinfected by placing them in a 70% ethanol aqueous solution for 3 min and thoroughly washing with sterile distilled water. After this first wash, the seeds were kept in a 2.5% sodium hypochlorite solution containing four drops of Tween 20 gel for 15 min and washed again with sterile distilled water [31]. Clean seeds were planted in a MS solution media complemented with 30 g L^−1^ saccharose plus 7.5 g L^−1^ agar and submitted to a 16 h light/8 h dark photoperiod cycle prior to sprout multiplication [14]. Leaf explants and stems were used to induce embryogenic callus formation in the previously described MS medium to which 0.5 and 1.0 mg L^−1^ of 2,4-D and 5 and 7 mg L^−1^ of ANA were added as callus formation inductors. Statistics were gathered according to a completely randomized experimental design with one category factor [14]. The soft part of the callus was cut away and placed in the MS solution with no agar and submitted to orbital shaking at 13,000 rpm for 30 min. The suspension was diluted 1:3 with water to minimize particle aggregation, and a subculture was started. Two weeks later, cells were counted in five samples for 5 days using the Neubauer camera. The turbidity of the resulting suspensions was measured using a field turbidimeter [22].

### 3.3. Application of the Analytic Method 

Cyclic voltammetry with a Pt working electrode was used as the analytical technique. As glutathione is one of the most abundant CRps in nature, it was chosen as the model peptide for this study. Oxidation current signals using the Pt electrode and GSH as a model were used to obtain the calibration curves for the peptide determination. Five different electrode cleaning procedures were used to select the best one for achieving the working curve with the best linearity coefficient: (1) no cleaning at all; (2) polishing with alumina powder, 0.5 μm, and ultrasound for 5 min; (3) ultrasound for 5 min plus CV treatment in a 0.5 mol L^−1^ H_2_SO_4_ solution between 0.1 and 1.15 V [32]; (4) ultrasound for 5 min plus CV treatment and subsequent bath in concentrated nitric acid for 5 min; and (5) ultrasound plus CV treatment and determination of blank signals in KI solution before any analytical measurement [33]. Calibration curves were plotted using the average value of the results of four consecutive measurements carried out after each electrode cleaning procedure. Repeatability of CRp measurement was determined by measuring a given sample three times during the same day, and reproducibility was checked by measuring a given sample three times in different days by different analysts. Detection and quantitation limits were calculated based on 10 consecutive measurements [26].

### 3.4. Measurement of Peptides in the Cell Suspensions

Suspensions were diluted 1:10 using MS media containing 0, 10, 50, and 100 µmol L^−1^ CdSO_4_. Each solution was left standing for 1, 2, 3, and 5 days after being washed with a 0.14 mol L^−1^ NaCl, 3 mmol L^−1^ KCl, 4 mmol L^−1^ Na_2_PO_4_, pH 7.4 solution, manually homogenized, and centrifuged at 13,000 rpm for 30 min. After separation of cell residues, the samples were ready for analysis [21]. A 2 mL sample supplemented with a 0.2 mol L^−1^ K_2_SO_4_ plus 1 mmol L^−1^ KI solution was analyzed using the standard addition method [18]. Under physiologically normal conditions, glutathione exists in its reduced form (GSH), while it changes to its oxidized form (GSSH) under stress conditions as a result of the route of elimination of reactive oxygen species, which can lead to cell death. In our experiments, we added approximately 2% sodium borohydride to the sample in order to transform all glutathione species into GSH and measured the total glutathione (t-GSH). Samples were neutralized with a 1 M HCl solution to pH 7 before analysis. [11].

## 4. Conclusions

Determination of CRp by cyclic voltammetry using Pt as a working electrode and a KI solution as supporting electrolyte constitutes a valid methodology, which provides detection and quantitation limits of 4.82 μmol L^−1^ and 15.41 μmol L^−1^, respectively. The use of this methodology for determination of CRp generation by *Solanum betaceum* cells and tobacco cells, both cultivated in vitro under the presence of increasing concentrations of Cd, showed that the *Solanum betaceum* cells accumulated cadmium with similar or better efficiency than tobacco cells. CRp generation kept a direct relationship with the amount of Cd(II) and the time to which the cells were exposed to it. *Solanum betaceum* cells behaved as good as the tobacco cells with respect to cadmium accumulation and usefulness as indicators of heavy metal contamination. Tamarillo cells offer the following advantages over tobacco cells: (1) a higher tolerance to the amount of contaminant they are exposed to; (2) longer exposure time before apoptosis, which means longer periods before replacement; and (3) faster stem growth. We are confident that the methodology proposed here will be very convenient and viable for field determination of CRp generated by some plants under contamination stress and therefore be very useful for detecting heavy metal pollution in crops. Strategic planting of *Solanum betaceum* seeds, along with other crops of interest, could be a more economic and less care-demanding strategy for indication of heavy metal contamination than using tobacco plants for the same purpose.

## Figures and Tables

**Table 1:** (*). Percentage of embryogenic callus formation of *Solanum betaceum*.

Medium	2,4-D (mg L^−1^)	ANA (mg L^−1^)	Stems		Leaves	
			% 1	% 2	% 1	% 2
1	0.5	0	70	65	10	10
2	1	0	90	95	0	10
3	0	5	30	40	50	50
4	0	7	10	10	25	30
		***p* value**	0.002		0.0011	

(*) Each treatment was done twice.

**Table 2:** Variation coefficient of repeatability and reproducibility tests and determination of the coefficient of linearity.

Parameter	Accepted *	Result
Repeatability	CV < 2%	1.57%
Reproducibility	CV < 3%	2.25%
Linearity	r^2^ > 0.98	0.9972

* Criteria described by Peters (2007) [26].
